# The paramount parameter: arterial oxygen tension versus arterial oxygen saturation as target in trials on oxygenation in intensive care

**DOI:** 10.1186/s13054-018-2257-9

**Published:** 2018-11-22

**Authors:** Olav Lilleholt Schjørring, Bodil Steen Rasmussen

**Affiliations:** 1 Department of Anaesthesia and Intensive Care Medicine, Aalborg University Hospital, Hobrovej 18-22, 9000 Aalborg, Denmark; 2 Department of Clinical Medicine, Aalborg University, Søndre Skovvej 15, 9000 Aalborg, Denmark

**Keywords:** Oxygen inhalation therapy, Hyperoxia, Hypoxia, Intensive care units, Respiratory insufficiency, Critical illness

## Main text

Oxygenation targets in critically ill patients admitted to the intensive care unit (ICU), in particular in patients with acute hypoxaemic respiratory failure, are still a matter of debate. There is mounting evidence for potential harm through hyperoxia [1–3]. Nevertheless, the optimal oxygenation targets, which minimise hyperoxia while maintaining sufficient oxygenation to avoid harm through hypoxia, remain unclear. Therefore, larger randomised clinical trials on the subject are needed. Several observational studies [4] as well as interventional before-and-after trials [1, 5] and three small randomised controlled trials [2, 3, 6] have added valuable, although not definitive, evidence to the field. Arterial oxygen saturation (SaO_2_) or pulse oximetry (SpO_2_) has been the primary parameter defining the target range in most of the interventional studies conducted. We would like to dispute this preference of SaO_2_ and SpO_2_ over arterial oxygen tension (PaO_2_) as the target parameter. Hence, this proposition for debate.

The general hypothesis of the studies on oxygen use in the ICU is that the dangers of oxygen toxicity are underestimated, and that the negative impact of hyperoxia is significant, even when compared to the risks of hypoxia following conservative oxygenation strategies [1, 2, 5, 6]. Hence, studies proposing more conservative oxygenation targets have been conducted primarily in the effort to avoid hyperoxia. The parameters PaO_2_ and SaO_2_ are linked as visualised in the oxygen dissociation curve [7]. The interval of PaO_2_ in ICU patients spans upwards from approximately 7.3 kPa (55 mmHg) [8–10]. In this area, the oxygen dissociation curve is rather flat [7] and covers only a small range of SaO_2_ values. The SaO_2_ range becomes even narrower with hyperoxaemic levels of PaO_2_ depending on its definition, which varies from over 13.3 kPa (100 mmHg) to over 64.9 kPa (487 mmHg) [4], while the corresponding SaO_2_ encompasses only four numeric values from 97% to 100%. This limits the control of hyperoxaemia if SaO_2_ is used as the target parameter of oxygenation. Furthermore, when SaO_2_ defines the oxygenation target in clinical trials, the narrow SaO_2_ spectrum will likely result in a larger risk of an overlap of PaO_2_ or SaO_2_ between the conventional and interventional study groups.

One could argue that the use of SaO_2_ over PaO_2_ offers the possibility to use the non-invasive measurement of oxygenation, SpO_2_. The correlation between SaO_2_ and SpO_2_ is generally high which makes SpO_2_ invaluable in the continuous monitoring and titration of oxygen supplementation in the ICU [11]. Nevertheless, SpO_2_ is unreliable as a measure of arterial oxygenation in patients with sepsis [12], in patients with use of vasopressors [13], in patients with high or low body temperature [11], and in patients with hypoxaemia [12, 13]. Therefore, SpO_2_ cannot be used in the ICU without intermittent measurements of SaO_2_ for comparison as remarkable differences above 4.4 percentage points [11] may occur. Furthermore, SpO_2_ has been shown inadequate in identifying and in quantifying hyperoxaemia defined as PaO_2_ above 16.7 kPa (125 mmHg) at SpO_2_ levels above 96% [14]. Thus, targeting normoxaemic oxygenation levels in the upper part of the normal reference interval by using SpO_2_ would inarguably lead to episodes of definitive hyperoxaemia. Moreover, at the steep slope of the oxygen dissociation curve [7], where the differentiation in SaO_2_ is the highest, SpO_2_ is also inadequate in correctly identifying the oxygenation level in the ICU as SpO_2_ above 94% is necessary to avoid a risk of having SaO_2_ below 90% [13]. This fact further narrows the spectrum of differentiation when using SpO_2_.

An argument for using SaO_2_ over PaO_2_ is that under normal, healthy conditions more than 98% of the transported oxygen is bound to haemoglobin [7]. Therefore, SaO_2_ in combination with the haematocrit or haemoglobin level represents the most direct parameter for expressing the amounts of oxygen actually carried in the arterial blood whereas PaO_2_ is only a secondarily derived parameter. However, the oxygen dissociation curve [7] shows that SaO_2_ and PaO_2_ are mutually dependent, and so this point remains essentially theoretical. Additionally, with increasing hyperoxaemia SaO_2_ loses its value due to the rigid ceiling of SaO_2_ at 100%. Furthermore, since the formation of reactive oxygen species is closely linked to the free amounts of oxygen [15] and since the reactive oxygen species contribute importantly to the detrimental effects of hyperoxia [15], PaO_2_ is probably the parameter with the tightest relationship to the toxic properties of oxygen. As these toxic properties are what trials on the subject strive to minimise, one could claim that this connection is just as important as the link between SaO_2_ and the total oxygen content of the blood.

In clinical practice, both SaO_2_ and PaO_2_ are commonly used to guide oxygen therapy, particularly in the ICU setting, as the majority of acute critically ill ICU patients require arterial cannulation for haemodynamic monitoring. For evaluating arterial oxygenation, a recent survey of northern European ICU physicians has shown that PaO_2_ was preferred to SaO_2_ [16].

In summary, to define the oxygenation target levels precisely, to reduce the risk of unwanted hyperoxaemia, and to minimise overlap between conventional and interventional groups, in clinical trials of higher versus lower oxygenation targets in the ICU population, PaO_2_ is in our opinion the superior target parameter as compared to SaO_2_. This is also reflected in clinicians’ self-reported preferences. Therefore, we advocate the use of PaO_2_ as the primary target parameter of arterial oxygenation in future clinical trials that aim to establish the evidence of how to use medical oxygen in patients admitted to the ICU.

## Abbreviations

HOT-ICU: Handling Oxygenation Targets in the Intensive Care Unit
ICU: Intensive care unit
PaO_2_: Arterial oxygen tension
SaO_2_: Arterial oxygen saturation
SpO_2_: Pulse oximetry
